# Worldwide Research Trends for Chelates in Animal Science: A Bibliometric Analysis

**DOI:** 10.3390/ani13142374

**Published:** 2023-07-21

**Authors:** Jalil Ghassemi Nejad, Reza Vakili, Ehsan Sobhani, Mahmood Sangari, Amir Mokhtarpour, Seyed Ali Hosseini Ghafari

**Affiliations:** 1 Department of Animal Science and Technology, Konkuk University, Seoul 05029, Republic of Korea; jalilgh@konkuk.ac.kr; 2 Department of Animal Science, Kashmar Branch, Islamic Azad University, Kashmar 7635168111, Iran; 3 Young Researchers and Elites Club, Mashhad Branch, Islamic Azad University, Mashhad 9177948564, Iran; 4 Department Library and Information Science, University of Birjand, Birjand 9717434765, Iran; 5 Special Domestic Animals Institute, Research Institute of Zabol, Zabol 9861335856, Iran; 6 The Agricultural Faculty, Agricultural Sciences and Resource Management in the Tropics and Subtropics (ARTS), University of Bonn, D-53115 Bonn, Germany

**Keywords:** chelate, intellectual structure, co-reference, keyword co-occurrence, hot topics, emerging topics

## Abstract

**Simple Summary:**

The application of chelated trace minerals (CTM) in livestock nutrition is an expanding research area with a focus on the health and quality of animal products. The aim of the current study was to conduct a scientometric analysis of the last three decades on CTM application in animal nutrition to point out main sources, most prolific countries, and current and emerging topics. This investigation is descriptive in terms of type and practical in purpose. The findings showed that the words zinc, copper, performance, pig, cattle, metabolism, and bioavailability were prominent words reflecting the hotspots in the chelate field during the investigated time period. The frequencies of the terms health, muscle, bovine, trace elements, and dietary supplements indicate that they are emerging topics in CTM as research has begun to focus on these areas.

**Abstract:**

The purpose of this study was to look at research trends in the application of CTM in animal nutrition in order to identify current and emerging challenges, as well as to examine the intellectual structure of the subject. The intellectual structure of CTM was examined using keyword and reference analysis. The research community includes all research and review articles published in journals indexed in the Web of Science database during the years 1990–2022. The results showed that the terms zinc, co-occurring 331 times, performance (324 times), and copper 216 (times) were the main and hotspots of research in the field of chelate. The data suggest that the most important keywords during the study period were zinc, copper, pig, bovine, metabolism, and bioavailability. The terms health, muscle, beef, trace elements, and dietary supplements represent emerging topics in CTM, as research began to focus on these areas during the years 2017–2022. The country with the greatest number of published articles was the United States of America. This bibliometric analysis showed that countries are focusing on the effects of CTM on the health and musculature of cattle through dietary supplementation with trace elements. According to the identified hot and emerging topics, this research can serve as a roadmap for a global comprehensive scientific plan and policy.

## 1. Introduction

In recent decades, the importance of trace elements in livestock and poultry production has been well documented [1]. Trace elements are among the natural needs of broilers and other domestic animals, and are added to their diets through mineral supplements [2,3]. Copper, selenium, iron, iodine, manganese, and zinc are the six elements in broiler diets that are given in the form of inorganic compounds (mainly oxides and sulfates). To increase absorption efficiency and reduce environmental impact, these elements are used in the form of organo–mineral complexes called chelated trace minerals (CTM) [4]. In biological systems, there are some types of organic mineral complexes, e.g., essential CTM for metabolism, such as vitamin B12, hemoglobin, or CTM, that interfere with the utilization of essential cations, such as zinc–phytic acid binding. Organic mineral complexes often consist of proteins and metal elements, and are effectively absorbed by animals, which brings many health benefits. The most important property of CTM is their high absorption and stability coefficient [5]. In fact, chelates of organic matter (especially proteins) and metal elements are effectively absorbed in the intestine and provide the opportunity not only to reduce the standard content of these minerals in the diet without adverse effects on the production characteristics of livestock and poultry, but also to reduce the environmental pollution caused by their cumulative aggregation [2,3]. Thus, there is an increasing interest in using organic forms of minerals compared to inorganic forms in animal nutrition. Given these properties, zinc and copper chelates are the best known of these products [6].

Previously, mineral research focused on the prevention of deficiencies in birds and domestic animals, and nutrient requirements were formulated accordingly. The complexity of mineral nutrition requires a complete study of performance, interactions, and resource availability by the producer and nutritionist at all times [7]. Microminerals play an important role in the metabolic processes of the body and are often involved in building catalysts, enzymes, and hormones, and their consumption would ensure health, growth, and fertility [8]. These minerals ensure proper growth, bone development, feathering of birds and growth of horns, skin, and hair in mammals, and also play a role in the structure and function of enzymes and hormones.

The insufficient supply of these substances affects many metabolic processes and causes various symptoms such as growth reduction, loss of appetite, reproductive defects, decreased immunity, general diseases and reduced the durability of meat and other livestock products. The deficiency of these elements is due to their insufficient amount in the diet or the presence of antagonistic substances that interact with their absorption [9].

Since the 1950s, some trace elements (copper, iron, iodine, manganese, molybdenum, selenium, and zinc) have been used in poultry diets [10]. These elements were formerly supplied through mineral compounds (mainly oxides and sulfates) and had no impact on ration prices due to their low cost. In the 1960s, new high-yielding lines of chickens and other livestock entered the market, and their nutrient requirements for all nutrients, especially micronutrients, had increased. High-yielding livestock requires high quality feed, and hence the bioavailability of minerals forms an essential component in the production system. In the last decades of the last century, microminerals were marketed in the form of chelates with organic substances, called “organic minerals”, “chelated trace minerals”, or “bioplexes”, which are in some respects better than primary mineral sources [11]. In these materials, the mineral element is bound to an organic substrate.

Scientometric analysis is a quantitative and statistical approach for assessing the structure and trends of scientific publications in a given field. In recent years, scientometric analysis has been used to identify current issues, emerging trends, and research gaps in various scientific fields. According to the scientometric assessment of the literature from the standpoint of research structure, many studies have been undertaken in various scientific domains to identify hot and developing issues in order to construct a scientific map [12,13,14,15]. Co-occurrence analysis of keywords has been used for animal models [16,17,18,19,20]. However, research on intellectual structure using two techniques, including co-occurrence analysis of keywords and reference analysis, is scant. As far as we know, there is currently no research addressing the latest developments and emerging trends in the use of CTM in animal nutrition. Despite the growing interest in the use of chelated microminerals in animal nutrition, there is a knowledge gap regarding the current trends and potential applications of CTM.

Therefore, this study utilizes keyword coincidence and reference analysis over a 32-year period to determine the trends and research gaps in CTM application in animal nutrition. The results of this analysis will provide valuable insights to researchers and industry professionals to address critical research areas in the use of CTM in animal nutrition. It should be noted that our paper’s focus was not on discussing specific findings, but rather on providing a comprehensive overview of the literature in this field.

## 2. Materials and Methods

### Description of the Procedures

This is a descriptive study using keyword co-occurrence and reference analysis. Data were extracted from the Web of Science (WOS) expanded scientific citation index on 10 January 2023. The literature search included all research and review articles and books on CTM from 1990 to 2022 in peer-reviewed journals indexed in WOS. To obtain data, the Web of Science Core Collection was selected. Then, in the advanced search of this database, the following combinations of formulas and keywords (Topic ((chelate) OR (micro mineral) OR (trace minerals) OR (organic minerals))) were used AND (ANIMAL SCIENCE) AND (LIMIT-TO (SUBJAREA) AGRI”) are used and confined to research and review articles. Finally, to make the data readable by the software CiteSpace (v. 5.8.R3, Philadelphia, PA, USA), VOSviewer (v. 1.6.17, Leiden, The Netherlands), and HistCite (v. 12.03.17, Philadelphia, PA, USA), the data were stored on a computer in plain text format in categories of 500 records (since Clarivate Analytics limits the number of downloaded records to 500). The retrieved files were then compiled into a single comprehensive file and analyzed. To appropriately generate and evaluate thematic maps, keywords were homogenized using a thesaurus, removing similar and identical keywords, as well as plural and singular forms of integration and non-specialized keywords.

## 3. Results

### 3.1. Annual Growth of Publications

**Figure 1:**
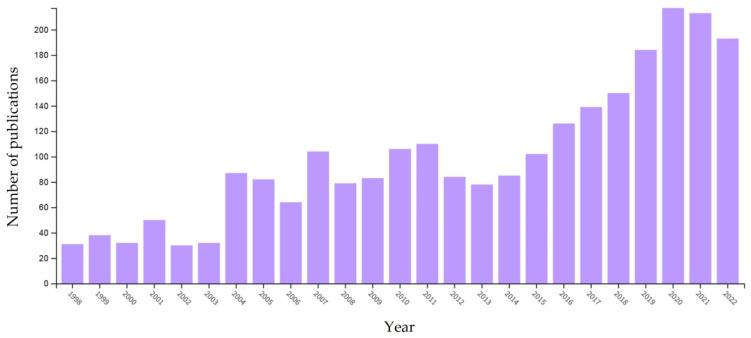
Trends in annual number of publications in the chelate field.

Over the three decades, the annual number of publications on in the chelate field exhibited a significant increase, with the highest number of articles (n = 217) being achieved in 2020. A total of 2853 articles were published between 1950 and 2022 relevant to CTM. The annual production of publications on chelates in the three decades investigated in our study rose steadily from ten documents in 1990 to 185 documents in 2022. The annual growth of publications was relatively slow from 1990 until 2000, and the average number of publications was 29 articles per year. The article publications ranged from 30 to 110 in the period from 2001 to 2011, with an average of 75 papers per year, which increased by about 2.6 times compared with the previous decade. Between the years 2012 and 2022, the average number of publications was 142 papers per year, and ranged from 78 to 217 (Figure 1).

### 3.2. Document Sources

**Figure 2:**
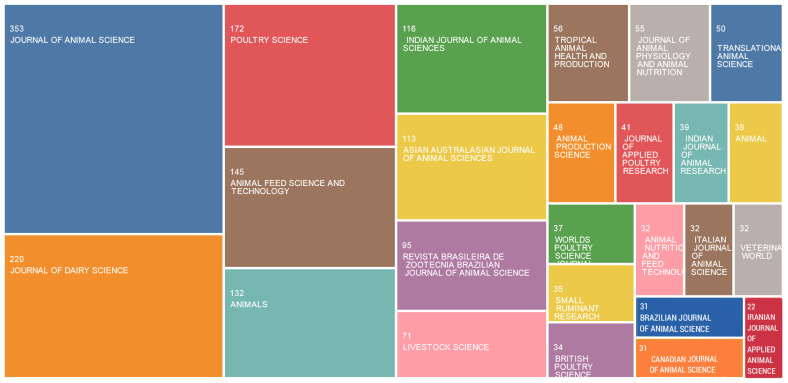
Journals contributing to the research in the chelate field.

A total of 191 journals were involved in the publication of articles in the chelate field. The top 25 journals with publications on chelates are given in Figure 2. In total, 2030 articles were published in these top journals. The highest number of articles published on CTM was found in the Journal of Animal Science, with 353 articles, thereby contributing 17.39% of the total papers, followed by the Journal of Dairy Science (n = 220), Poultry Science (n = 172), Animal Feed Science and Technology (n = 145), and Animals (n = 132). Among these top 25 journals, the Journal of Dairy Science had the highest impact factor (4.225), and 56% of these journals were partitioned into JCR Q1, and Q2. This may indicate that hot topics and future trends on chelated minerals in farm animals are likely to be reported in these journals.

### 3.3. Publishers Contribution

**Figure 3:**
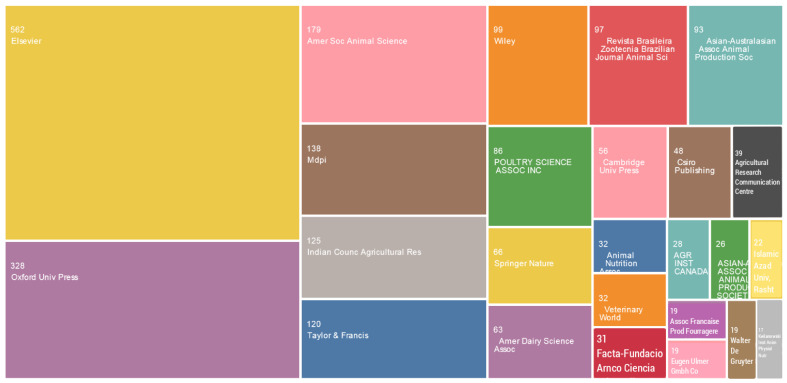
Top 25 publishers to publish on the topic of chelates.

The top 25 publishers to have published articles on topics related to CTM are presented in Figure 3. A total of 135 publisher records were identified between 1951 and 2022. The greatest number of articles published on CTM was in Elsevier, accounting for 19.7% of total published papers. Of the 1827 articles published by the top 10 publishers, 562 articles were published by major publishers, namely Elsevier, followed by Oxford University Press (n = 328), the American Society of Animal Science (n = 179), MDPI (n = 138), the Indian Council of Agricultural Research (n = 125), Taylor & Francis (n = 120), Wiley (n = 99), Revista Brasileira De Zootecnia—Brazilian Journal Of Animal Science (n = 97), the Asian–Australasian Association of Animal Production Societies (n = 93), and Poultry Science Association Inc. (n = 86).

### 3.4. The Intellectual Structure and Network of the Chelate Field

**Figure 4:**
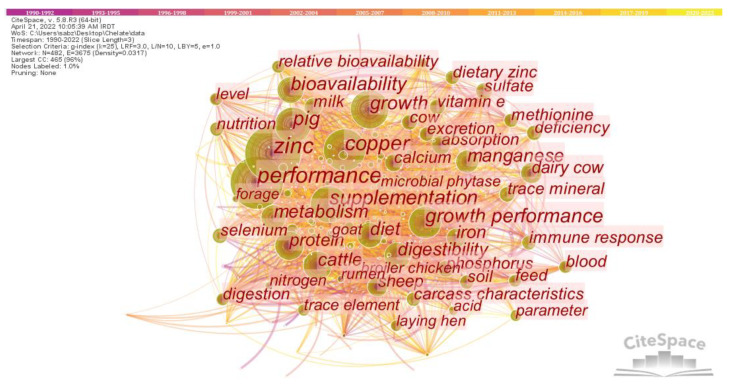
The intellectual structure and network of the chelate field.

Figure 4 provides an overview of the intellectual structure and network of the chelate domain and illustrates the relationships and connections among the various nodes. The size and color of each circle in the network indicates the frequency of encounters and the time period in which they originally occurred. The network consists of 428 nodes and 3685 links, with a network density of 0.317. The lines connecting the nodes illustrate the links between different research topics and the relationships between them. The colors of these lines indicate the time when these connections were first made [21]. From the network, it can be seen that important terms such as zinc, copper, performance, pig, cattle, metabolism and bioavailability were identified as hotspots in the field of CTM during the analyzed period. Analysis of Table 1, which provides detailed information on each time period, shows that terms such as zinc, performance, copper, and supplementation were the most frequently discussed topics in chelate research. These topics mostly date from between 1991 and 2008.

**Figure 5:**
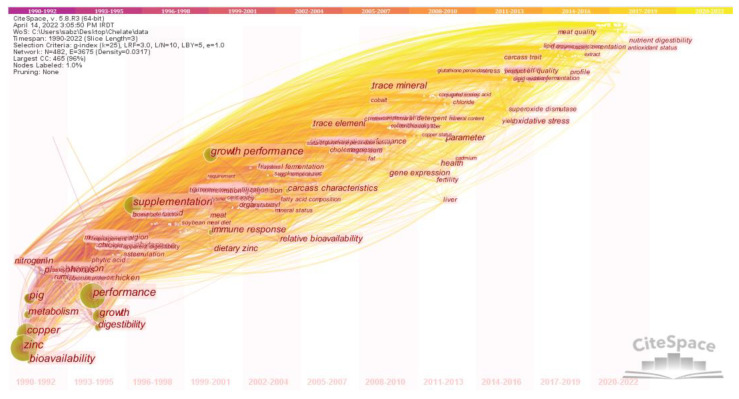
Keyword co-occurrence map based on time periods.

The co-occurrence of keywords related to chelate over time was analyzed in Figure 5. The figure is divided into time zones, with nodes and links moving from left to right, indicating the evolution of research interests in chelates over time. The figure shows that certain keywords were more popular during certain time periods. For example, from 1990 to 1995, pig, metabolism, copper, bioavailability, performance, growth, and digestibility were in frequent use. From 2001 to 2008, research topics shifted to growth performance, trace element, cobalt, dietary zinc. In the most recent time period (2010–2019), new research topics include carcass traits, meat quality, nutrient digestibility, and antioxidant status.

### 3.5. Keywords with the Strongest Citation Bursts

**Figure 6:**
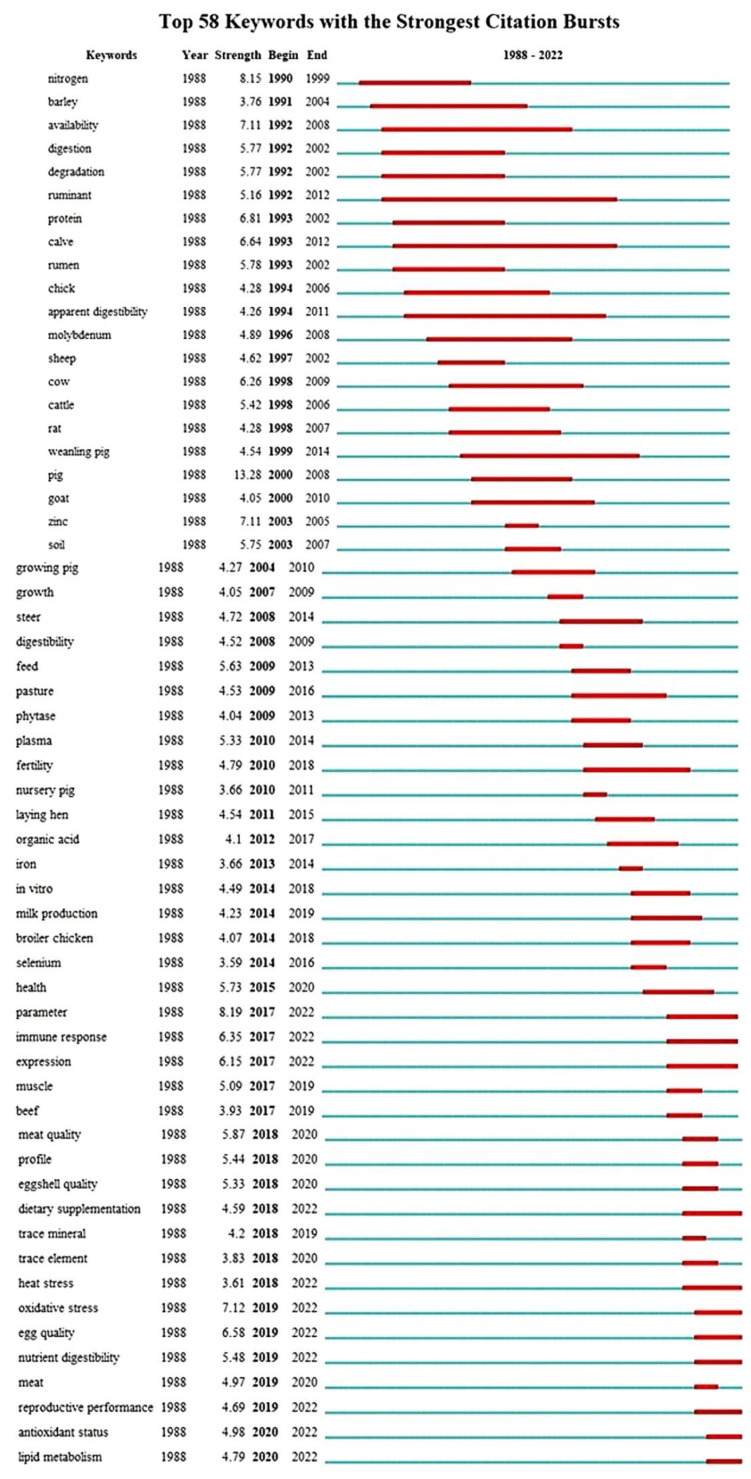
Top keywords based on the strongest citation bursts.

Figure 6 shows the 58 keywords that experienced the most significant citation bursts over the years, and the time periods during which these topics were studied. The lines in the graph are color coded to indicate different time intervals. The blue lines indicate the years in which a particular topic was studied, with a decreasing trend in its consideration over time. In contrast, the red lines represent the beginning and end of a topic’s burst and indicate its hotness at a given point in time. It can be seen from the graph that most of the red lines occur near 1988, indicating a period of intense interest in chelate research. The blue lines, on the other hand, are concentrated around 2022, indicating recent and ongoing studies in the field. The figure suggests an evolutionary progression of topics from 1988 to 2022. The most frequently cited keywords, undergoing strong citation bursts, include minerals, organic minerals, trace elements, and metal-binding peptides. This information provides insight into the areas that have attracted the most attention in chelate research over the years and can serve as a basis for future studies.

The strength or intensity of a keyword in Figure 6 indicates the degree of attention and importance given to that topic in each period. It is noteworthy that the keywords “availability”, “ruminant”, and “calves” have the longest burst time, indicating that these topics were of interest to the scientific community for 28, 19, and 16 years, respectively. The keyword “pig” was cited the most during the period 2000–2008, with a citation rate of 13.28. The hotspots and emerging topics in the field of CTM can be divided into three phases, based on the timeline shown in Figure 6. The first phase spans 1990 to 2006, when the research hotspots were nitrogen, barley, protein, sheep, and zinc. The second phase spans 2007 to 2016, during which period the hotspots included pasture, plasma, selenium, and so on. The third and final phase covers the period from 2017 to 2022, and focuses on meat quality, antioxidant status, health, muscle, beef, trace minerals and supplements, among others.

**Figure 7:**
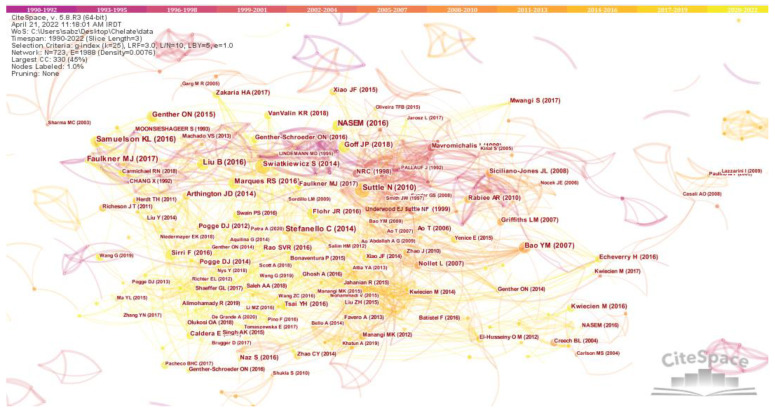
Keyword co-occurrence map based on a timeline diagram (prominent researchers).

The author map shown in Figure 7 provides insight into the most prominent researchers in the field of CTM, determined on the basis of the co-occurrence of keywords and the timeline of their studies. The map consists of 723 nodes and 1988 links, with a network density of 0.0076. The lines connecting the nodes move from left to right, indicating the chronological evolution of the authors’ research interests [22]. The map highlights several significant researchers who have made major contributions to the field of CTM. These prominent researchers include the authors cited in references [23,24], each of which has made significant contributions to the research on CTM. The research interests of these prominent researchers evolved over time. Some researchers focused on topics such as animal nutrition, trace elements, and dietary supplements, while others focused on topics such as mineral bioavailability and the development of chelated compounds.

Table 2 presents a list of 24 references with more than 10 citations published between 1999 and 2018. Notably, the most frequently cited reference was [23], which has been cited by 20 studies, followed by the study presented in [25], with 18 citations.

### 3.6. Co-Reference Citation Network with the Strongest Citation Bursts

**Figure 8:**
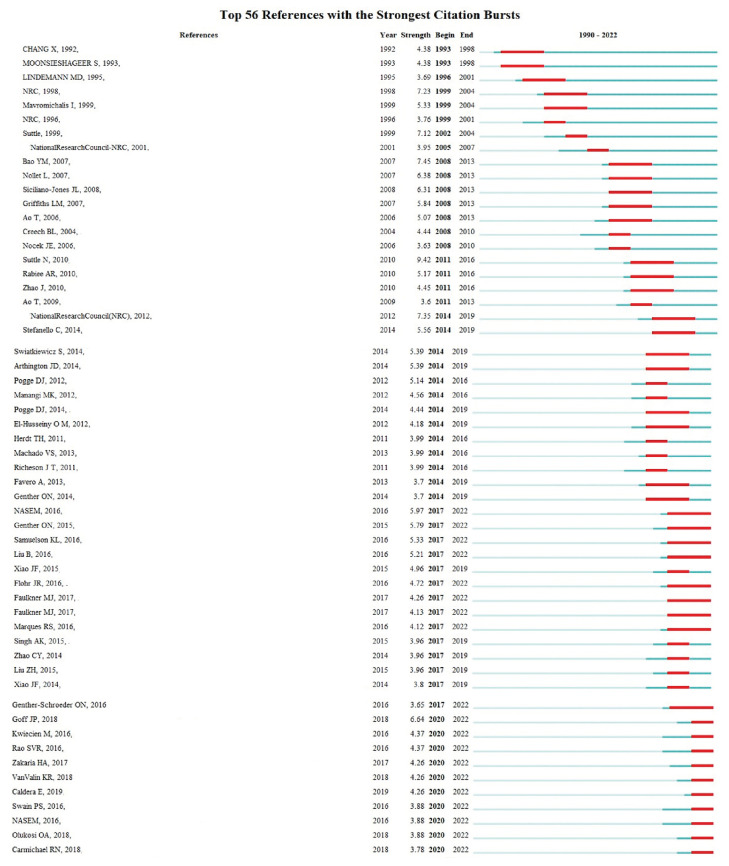
Top references on the basis of the strength of their citation bursts [23,26,47,48,49,50,51,52,53,54,55,56,57,58,59,60,61,62,63,64,65,66,67,68,69,70,71,72,73].

Emerging trends can be seen by considering the articles that have received the most citations in recent years [46]. Figure 8 shows the most cited articles in the field of CTM. It is clear that the increase in citations began in 2020 and continued through 2022, indicating that these articles are attracting significant attention. These articles highlight emerging trends and hot topics in the field of CTM, and it is likely that they will continue to receive significant attention in the future.

**Figure 9:**
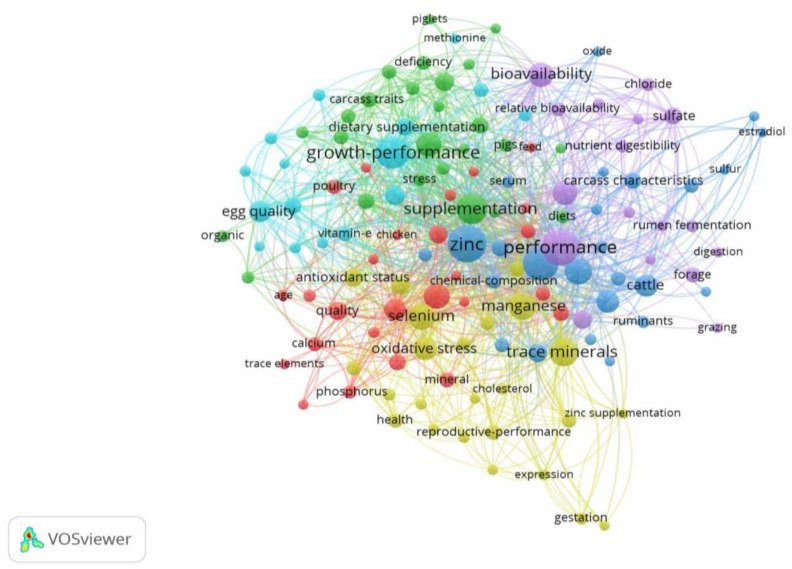
Concepts and research topic clusters in the chelate field.

In contrast to the other tables and figures, which quantitatively evaluate topics in the field of CTM, Figure 9 presents the concept map of studies in the CTM research area. It is worth noting that the size of the circles indicates the extent to which these concepts are used in characterizing the articles, and their color indicates the clustering of the concepts. In addition, the distance and proximity of the keywords in the map show how closely related the concepts are. Nodes with the same color indicate synergistic effects between them. For example, as can be seen in Figure 9, zinc supplementation can affect cholesterol, health and reproductive performance. This information could assist researchers in designing their studies more effectively and investigating the interrelationships among various factors.

**Figure 10:**
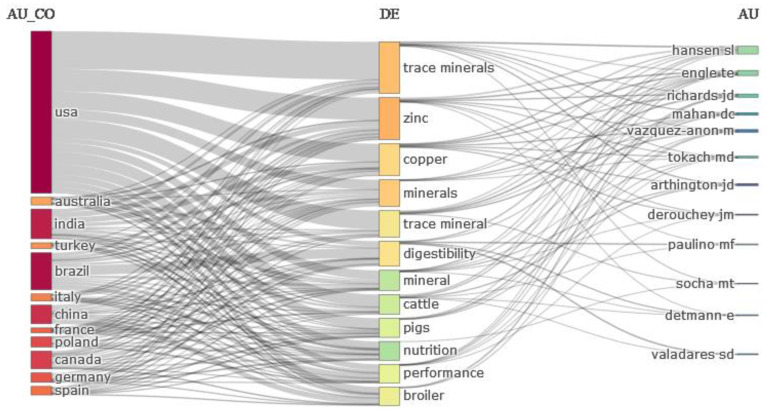
Three-field plot for the relationships among authors, countries, and author keywords in chelate research. (AU: author; Au_CO: author country; DE: document keyword).

Figure 10 is a visual representation consisting of three panels that depict the relationships among authors, countries, and keywords in chelate research. The rectangles in the diagram represent each entity: authors on the right, countries on the left, and keywords in the center. The height of the rectangles corresponds to the number of studies conducted by/in each country, author, and research area. The more studies associated with an entity, the larger and higher the rectangle. The communication lines in the diagram indicate the links between authors and countries. The strength of the connections between the entities is directly proportional to the amount of research conducted by the authors and countries on the topic. In other words, the stronger the connections, the greater the amount of research conducted by authors and countries. Overall, Figure 10 provides a clear picture of the distribution of research activities among different countries, authors, and research areas in the field of chelates.

This analysis of chelate research revealed that some countries have conducted more studies on this topic than others. In particular, it was found that the United States of America, Australia, India, and Turkey have performed the most research, as indicated by the height of the respective rectangles in the diagram presented in Figure 10. A closer examination of research conducted in the United States revealed that the majority of studies were centered around trace elements, specifically, zinc, copper, and other minerals. Similarly, most of the studies conducted in Australia were focused on topics such as trace elements, copper, cattle, and swine, and were mainly conducted by the researchers mentioned in references [74,75,76,77]. On the other hand, the studies conducted in India and Turkey also focused on trace elements, zinc, copper, and minerals.

## 4. Discussion

### 4.1. Annual Increase in the Number of Publications

The present study, despite having some limitations (e.g., the database used and the different methods and indicators employed), is the first bibliometric analysis in the field of chelated trace minerals. This study provides interesting information on the evolution and development of research in this area. The study identifies the topics that are considered basic knowledge in this field, the research areas that are currently being neglected, and the emerging topics that are currently being studied by scientists.

Several researchers from different countries have collaborated on studies to address chelate administration in livestock and poultry. This necessitates a bibliometric analysis in order to be able to understand the milestones achieved and the future research trends. In recent decades, there has been an increasing recognition of the role of minerals in health, which has led to a growing interest in research on the application of chelates in farm animals, particularly in cattle and pigs. This is demonstrated by the increasing number of publications appearing each year, which serves as a measure of interest in this area of research [78,79,80].

An increasing knowledge of the impacts of chelated minerals on animals and their association with health [6,81,82] is evidenced by the increase in the number of research trends between 2012 and 2022 [83,84], as can be seen from the results for the strongest citation bursts. Over the last five years, there has been an increase in the annual production of papers by 189 papers, corresponding to 35.3% of the total. Hence, in order to increase the annual number of publications, growth rate, and number of citations of articles on CTM and health, efforts must be made to further boost research and collaboration. The increase in average annual citations is positively correlated with the increase in the number of publications, exhibiting a monotonic connection. Furthermore, it is anticipated that the average number of citations will keep rising as the number of citable years rises. Therefore, to increase the annual number of publications, growth rates, and number of citations of articles on chelates and farm animals, consistent efforts to promote research and collaboration are crucial.

### 4.2. Document Sources and Publisher Contributions

The CTM-related subjects that attracted a lot of interest in the assembled literature offer helpful insights into the top research goals in journals. Conversely, areas that received little attention offer opportunities for additional study and advancement in this area. Researchers can expand our knowledge and improve our understanding of CTM by looking into and addressing these less thoroughly explored topics. This may result in new understandings, uses, and enhanced use and efficiency of CTM. One of these areas is biochemistry and molecular biology. Additionally, the different approaches taken by journals to CTM may help to dispel the myth that publishers do not regard this idea to be significant. This misunderstanding could also be due to the huge variations in how journals manage their progress. The Journal of Animal Science has the largest network, and a tight association with other journals, including the Journal of Dairy Science, Poultry Science, Animal Feed Science and Technology, and Animals, according to an examination of journal co-citations and bibliographic coupling. Due to the publication of influential discoveries in these publications, it is most likely that these journals will report on new themes and future trends in chemical research in animal science. Notably, articles from the Journal of Animal Science make up the majority of referenced articles worldwide, thus demonstrating the reliability, dependability, and authority of this journal as a source of high-quality research and knowledge about the production of poultry.

The focus on CTM in the above-mentioned journals can be better understood in light of the results of this study, making it easier to identify the goals, shared advantages, and prospects for industrial partnerships among journals. This study also identifies areas where international collaborations could advance CTM research, such as by examining the advantages and disadvantages of using various farm animal breeds or by examining nutritional digestibility. It also highlights the significant research and development capabilities of scientists. Bibliometric analysis, which includes looking at journals and books, is extremely important for delivering the most recent insights and trends in certain fields of study, and helps researchers choose the best journals in which to publish their research communications [85].

Publishing the highest number of articles in the chelate field in Elsevier was not unexpected, as it publishes more than 2800 digitized journals, and 46,000+ eBook titles. Moreover, Elsevier offers a range of pay-to-read and pay to-publish options, both subscription-based and transactional, to fit the diverse needs of institutions, funders and researchers worldwide [86].

### 4.3. The Intellectual Structure and Network of the Chelate Field

An analysis of the intellectual structure and network of the chelate field provides a comprehensive understanding of the research trends and interrelationships among the scientific domains related to CTM. Compared to inorganic forms of trace minerals, such as sulfate and oxide, chelated mineral complexes contain ligands with carbohydrates, lipids, amino acids, and proteins [87]. This difference results in better mineral absorption in the chelated form, which can occur in any part of the small intestine, while inorganic minerals are typically absorbed in the duodenum [88]. Among trace minerals, zinc is considered to be the second most abundant mineral and serves as a co-factor in over 300 enzymes with respect to their structure or metabolic and catalytic activity [89]. Copper is another essential microelement, and is present in many enzyme systems in the body, such as superoxide dismutase, cytochrome oxidase, and lysyl oxidase, or ceruloplasmin [40], where it serves as a cofactor. Therefore, zinc and copper play crucial roles in various metabolic functions, making them important keywords in the field of chelated trace minerals (CTM). Although most studies have shown positive effects of organic minerals compared to inorganic minerals in both poultry and ruminants, the results are not always consistent, especially in terms of performance indices. For instance, some studies have reported no effects of dietary organic zinc supplementation on growth performance in broilers [90,91], laying hens [92], and lactating cows [93]. In-depth information on the function of microminerals as enzyme cofactors in limiting free radicals and their significance in the body’s antioxidant capacity was provided by Goff [30]. However, consuming too much of these minerals might have pro-oxidant effects and provoke detrimental free radicals. For organisms to maintain homeostasis, a precise balance between mineral toxicity and deficiency must be maintained. This can be achieved by increasing absorption or excretion. Given that they can contribute to the eutrophication of natural waters and environmental pollution, the release of minerals from undigested feed and urine excretion must be considered carefully when formulating feeds [94]. In summary, the intellectual structure and network analysis shown in Figure 4 and Table 1 provide insights into hotspots and emerging trends in the field of CTM. These results could inform future research directions and identify potential research gaps in the field of animal nutrition.

The map of keyword co-occurrence was created to reflect the scientific community’s interest in various research topics over time. The nodes in the figure represent research topics, and the lines connecting the nodes reflect the relationships between the topics over time, showing the emergence of research topics. The color of the clusters represents the emergence of a cluster and illustrates the evolution of research interests in the field of chelate. The shift in research topics from growth and performance in the 1990s to meat quality and antioxidant status in 2010s suggests that increasing numbers of researchers were gradually becoming involved in studies addressing the quality of meat and antioxidant status in farm animals. Several studies have been performed demonstrating the protective and beneficial effects of CTM on animal production, health and, eggshell quality [95], enriched egg content [96] and other metabolic variables in animal nutrition, such as acid–base [30] and antioxidant status [30,96,97]. Therefore, this intellectual structure and network analysis provides insight into the evolution and emerging trends in chelate research and allows researchers to identify key research areas and potential gaps in current research.

### 4.4. Keywords and Co-Reference Citation Network with the Strongest Citation Bursts

There were fifty-eight keywords that experienced the most significant citation bursts over time period in which these topics were studied. The use of keywords in research papers helps to express the material covered, promote the creation of new knowledge, speed up the retrieval of pertinent data, and draw attention to the relationships between various lines of research in a given field. In order to summarize the important ideas covered in their research, authors frequently include author-generated keywords when submitting an article. Furthermore, Web of Science generates Keyword Plus based on words and phrases found in the titles of referenced sources [98]. The explicit communication of the knowledge structure, the article’s content, and the interconnection of research in a particular topic is facilitated by the use of both author keywords and Keyword Plus. It is interesting to note that the keywords “availability”, “ruminant”, and “calves” had the longest burst times, showing that these subjects have attracted a lot of attention from academics and have gradually increased in importance in the studies [98,99].

The three phases of hotspots and emerging topics in the field of CTM show how the focus of research in CTM has shifted and evolved over time, reflecting the changing interests and priorities of the scientific community. The map of authors also shows collaborations and co-authorships between researchers in the field of CTM. The clustering of nodes indicates groups of authors who have collaborated on research projects and published together on similar topics. By analyzing the co-occurrence of keywords among authors, the map can provide insights into trends and developments in chelate research and collaborations that have led to significant contributions in the field. The study of CTM in animal science has benefited greatly from the United States’ contributions. The US tops the list for publications, major contributors, financial resources, institutional affiliations, and corresponding authorship. The most active university was Iowa State University in the US, while the Zinpro Corporation has made the most contributions to chelates in animal research in terms of publications and citations. In terms of articles and citations, Stephanie L. Hansen from Iowa State University has contributed the most to the use of chelates in animal nutrition. The most influential and productive journal in this area was the Journal of Animal Science [100]. According to network research, the US has also developed substantial partnerships with nations like Turkey, Australia, and India in terms of co-authorship, citations, and bibliographic coupling. Additionally, the US has the most networks and connections, and the highest total link strength (TLS), with other nations, demonstrating substantial cooperation. This could be due to the significant funding commitments made by the US government to fund chelate-related animal feeding research [78].

## 5. Conclusions

Our bibliometric analysis provided a thorough and in-depth evaluation of the previous three decades’ worth of global CTM research in animal science. This study is a useful resource for future investigations into the effects of chelates on animal science around the world. According to this study, there has been an increase in interest in CTM, as demonstrated by the significant increase in the number of published articles. The analysis indicates the frequent co-occurrence of certain keywords, such as zinc, performance, and copper, highlighting their significance in CTM research. Zinc and copper play crucial roles in various metabolic functions, making them important keywords in the field of CTM. The research topics shifted from growth and performance in the 1990s to meat quality and antioxidant status in the 2010s, which highlights a need for more focus and research on the development of these subjects to address the health issue. The United States contributed the highest number of publications and citations on the application of CTM in animal nutrition, and the Journal of Animal Science was the most productive and most cited journal. It seems that collaboration within and between authors from different countries in the field of CTM can be an important key to bridging the gap between the increasing global population and the production of high-quality and efficient animal feed. However, further large-scale studies are required to be able effectively utilize CTM in commercial animal production. Future studies on CTM may find inspiration in our findings as a starting point. We specifically want to encourage industries, academics, and institutions to concentrate their efforts on research hotspots or areas of significant research interest.

## Figures and Tables

**Table 1:** Top keywords in the chelate field based on the most co-occurrence.

Count	Years	Keywords (Topics)
331	1991	zinc
324	1993	performance
216	1990	copper
180	1996	supplementation
166	2004	growth performance
157	1993	pig
144	1995	growth
127	1991	bioavailability
121	1992	cattle
116	1992	digestibility
109	1994	manganese
109	1992	diet
105	1990	metabolism
101	1990	protein
82	1992	dairy cow
82	1990	iron
80	2000	quality
75	1991	absorption
74	1993	sheep
73	1991	calcium
69	1992	cow
65	2008	trace mineral
63	2002	deficiency
63	1994	sulfate
63	1994	phosphorus
61	1991	milk
60	2001	vitamin E
59	2007	selenium
58	2008	immune response
57	1993	excretion
56	1991	methionine
56	1990	soil
55	2002	dietary zinc
55	1995	feed
55	1992	digestion
55	1993	blood
52	1999	nutrition
51	2003	carcass characteristics

**Table 2:** Top references in the chelate field based on the number of citations.

Count	Year	Cited Reference
20	2016	[23]
18	2010	[24]
16	2016	[26]
15	2016	[27]
15	2014	[28]
15	2017	[24]
14	2007	[29]
14	2018	[30]
14	2015	[31]
14	2016	[32]
13	2014	[33]
13	2014	[34]
12	2014	[35]
12	2016	[36]
12	2007	[37]
11	2016	[38]
11	2016	[39]
11	1999	[40]
11	2008	[41]
11	2007	[42]
10	2010	[43]
10	2016	[44]
10	2015	[45]

## Data Availability

The data that support the findings of this study are available from the corresponding author upon reasonable request.
